# Genomic Amplification of an Endogenous Retrovirus in Zebrafish T-Cell Malignancies

**DOI:** 10.1155/2012/627920

**Published:** 2012-06-13

**Authors:** J. Kimble Frazer, Lance A. Batchelor, Diana F. Bradley, Kim H. Brown, Kimberly P. Dobrinski, Charles Lee, Nikolaus S. Trede

**Affiliations:** ^1^Department of Pediatrics, University of Utah, Salt Lake City, UT 84112, USA; ^2^Department of Oncological Sciences, Huntsman Cancer Institute, University of Utah, Salt Lake City, UT 84112, USA; ^3^Department of Pathology, Brigham and Women's Hospital, Boston, MA 02115, USA

## Abstract

Genomic instability plays a crucial role in oncogenesis. Somatically acquired mutations can disable some genes and inappropriately activate others. In addition, chromosomal rearrangements can amplify, delete, or even fuse genes, altering their functions and contributing to malignant phenotypes. Using array comparative genomic hybridization (aCGH), a technique to detect numeric variations between different DNA samples, we examined genomes from zebrafish (*Danio rerio*) T-cell leukemias of three cancer-prone lines. In all malignancies tested, we identified recurring amplifications of a zebrafish endogenous retrovirus. This retrovirus, ZFERV, was first identified due to high expression of proviral transcripts in thymic tissue from larval and adult fish. We confirmed ZFERV amplifications by quantitative PCR analyses of DNA from wild-type fish tissue and normal and malignant *D. rerio* T cells. We also quantified ZFERV RNA expression and found that normal and neoplastic T cells both produce retrovirally encoded transcripts, but most cancers show dramatically increased transcription. In aggregate, these data imply that ZFERV amplification and transcription may be related to T-cell leukemogenesis. Based on these data and ZFERV's phylogenetic relation to viruses of the murine-leukemia-related virus class of gammaretroviridae, we posit that ZFERV may be oncogenic via an insertional mutagenesis mechanism.

## 1. Introduction

Zebrafish are an emerging animal model for the study of lymphocytic cancers. A landmark 2003 study first described that transgenic murine *Myc* (*mMyc*) misexpression could induce *D*. *rerio* T-cell acute lymphoblastic leukemia (T-ALL) [1]. Since that initial report, several other zebrafish ALL models have been described, utilizing transgenic mammalian *TEL-AML1* (human), *NOTCH1* (human), *MYC* (murine and human), and *AKT2* (murine) in similar fashion [2–5]. In addition, we used a phenotypic mutagenesis screen to create three further zebrafish models with heritable T-ALL predisposition [6]. All but one of the eight lines cited above are prone to T-ALL, not B-cell-lineage cancers. Like human T-ALL, *D. rerio* T-ALL often arises in or spreads to the thymus and forms tumors. Hence, these seven zebrafish lines actually more accurately model two related lymphocyte malignancies, T-ALL and T-cell lymphoblastic lymphoma (T-LBL). In fact, *mMyc* zebrafish have even been used to investigate the molecular changes that accompany the transition between T-LBL and T-ALL [7].

Because the molecular origins of T-ALL and T-LBL are not completely understood, these zebrafish models provide opportunities to investigate the genetic underpinnings of these diseases' oncogenesis. In addition, they also facilitate inquiries designed to reveal features associated with T-ALL and T-LBL progression. For example, in the aforementioned study, Feng et al. demonstrated that changes in BCL2, S1P1, and ICAM1 expression were linked to autophagy, intercellular adhesion, and intravascular invasion, thereby governing the T-LBL to T-ALL transition [7]. Similarly, Gutierrez et al. used transgenic zebrafish T-ALL to study the dependence of MYC-driven cancers upon *Pten* and *Akt *for disease persistence and progression [5].

While these two studies utilized *D. rerio* models to investigate candidate genes of suspected importance to disease progression, zebrafish T-cell cancers can also serve as a means for candidate gene discovery. In our own work, we utilized serial allo-transplantation of *D. rerio* T-ALL as an experimental approach to model clinically aggressive neoplasia [8]. Similar strategies have been employed by other groups, using serially allo-grafted murine T-cell lymphomas or xeno-transplanted human T-ALL into immunodeficient mice [9, 10]. In our study, we performed aCGH to seek acquired genomic changes common to serially passaged *D. rerio* T-ALL and refractory/relapsed human T-ALL. Several candidate genes met this criterion, including *C7orf60* (zebrafish homologue, *zgc*:* 153606*), a gene whose amplifications were linked to both accelerated T-ALL progression in fish and inferior outcomes in human T-ALL patients [8].

Although our study concentrated on acquired copy number aberrations (CNAs) shared by zebrafish and human T-ALL, we also identified other genomic amplifications and deletions seen only in *D. rerio* cancers. Amongst these, two recurring copy number gains were observed in every sample, and with further scrutiny we found that both regions corresponded to the same endogenous retrovirus (ERV). This genomically integrated provirus is predicted to have 2–4 integration sites in the zebrafish genome [11], and its ploidy and genomic positions may vary between individual animals, complicating its inquiry. In this paper, we use two independent methodologies to show that this multicopy ERV undergoes further amplification in both normal and neoplastic zebrafish T cells, which could create new and potentially oncogenic integrations. Some cancers showed very high ZFERV copy number, well above that seen in T lymphocytes. We also demonstrate the expression of retrovirally encoded RNAs by both normal and cancerous *D. rerio* T cells, with most malignancies displaying significantly elevated proviral transcription relative to normal T cells. Our findings, and further characterization of this ERV, will be essential to understanding how this biologically active retrovirus impacts normal and malignant zebrafish T lymphocyte biology.

## 2. Materials and Methods

### 2.1. Zebrafish Lines and Care

Adult fish from five *D. rerio* lines were analyzed: normal WIK strain *lck*::*EGFP *fish [12], the ENU mutant lines *hulk*, *shrek*, and *oscar the grouch* (*hlk*, *srk*,* otg*; all WIK background) [6], and *rag2*::*MYC-ER *× *lck*::*EGFP *fish (*nacre* × WIK hybrid) [5]. Fish were housed using standard conditions (28.5°C, 14 hr. light/10 hr. dark circadian cycle) in a colony at the University of Utah's zebrafish core facility. For examinations under fluorescent microscopy, fish were anesthetized with 0.02% tricaine methanesulfonate (MS222) and euthanized with ice water prior to dissections. Animals were handled according to NIH guidelines, under an approved protocol (IACUC #08-08005) by the University of Utah Animal Care and Use Committee.

### 2.2. Dissections and Fluorescence-Activated Cell Sorting (FACS)

Zebrafish thymi and GFP^+^ tumors were dissected, with preparation of single cell suspensions and FACS performed as described previously [6]. BD FACSVantage and FACSAria II SORP (Becton Dickson) instruments were used for FACS. GFP intensity and side- and forward-scatter were gating parameters for GFP^+^ lymphocyte collections.

### 2.3. Nucleic Acid Purifications

Genomic DNA for aCGH and qPCR was extracted from FACS-purified GFP^+^ T cells and matched tailfin tissue using the DNeasy Blood and Tissue Kit (Qiagen) as described previously [8]. Total RNA for qRT-PCR assays was extracted from FACS-purified T lymphocytes and T cell cancers with Trizol (Invitrogen) or the RNeasy Mini-Kit (Qiagen) according to manufacturer instructions. RNA samples were treated with RNase-Free DNase (Qiagen) according to manufacturer instructions prior to qRT-PCR.

### 2.4. Array Comparative Genomic Hybridization (aCGH)

Genomic DNA was labeled with the BioPrime Labeling Kit (Invitrogen), purified, quantified, and hybridized to Zv6-based Zebrafish Genomic Arrays (NimbleGen) as reported previously [8]. Arrays were analyzed using the G2565CA Microarray Scanner System with SureScan High Resolution Technology (Agilent) and normalized using Agilent Feature Extraction software. Copy-number analysis was conducted using the Rank Segmentation algorithm with Nexus Copy Number 5.0 software (BioDiscovery). Detailed descriptions of the aCGH methods used and copy number analyses performed are available in the supplemental sections of the report by Rudner et al. [8].

### 2.5. Quantitative Polymerase Chain Reactions (qPCR)

A LightCycler CFX96 (Bio-Rad) was used for qPCR assays. Briefly, IQ SYBR Green Supermix (Bio-Rad) was used to amplify genomic DNA from various tissue types. Pooled thymocyte DNA (our limiting sample) was spectrophotometrically quantified and then diluted 1 : 100 for use in qPCR. DNA from tailfin tissue and GFP^+^ tumor cells were diluted to identical concentrations, with 2 *μ*L of each DNA used in reactions with total volumes of 25 *μ*L, and other components added according to manufacturer instructions. All reactions were performed in triplicate. SYBR Green signals were used to derive estimates of relative ZFERV copy number. Since true ZFERV copy number is unknown, values were arbitrarily normalized to 1 copy/haploid genome. Thus, a ZFERV relative copy number equal to 3 indicates three times as many ZFERV copies/genome (e.g., if germline copy number = 3 copies/haploid genome, ZFERV relative copy number = 3 indicates 9 copies/haploid genome). All qPCR results with *pol* and *env* were normalized to *elf2a*, present in 1 copy/*D. rerio* haploid genome. Primers and reaction conditions were as follows:

Forward *pol* primer: CGC-CCC-ACA-CAT-CAC-ATA
Reverse *pol* primer: CAA-CCA-TCA-CAG-AAC-AGA
Forward *env* primer: ATG-TTT-GGG-GAA-TGG-AAG-G
Reverse *env* primer: TTT-GAT-AAG-GAG-GTG-GGT-TTT
Forward *elf2a* primer: TGG-AGG-TGG-AGG-TGA-GAA-CT
Reverse *elf2a* primer: GAG-TGG-TTG-TGT-AAG-CAT-TTC-G
Denaturation: 95°C × 3 minutes
40 cycles:
  95°C × 10 seconds
  59°C × 40 seconds
Melt curve analysis—55°C–95°C.

### 2.6. Quantitative Reverse Transcription Polymerase Chain Reactions (qRT-PCR)

Total RNA (200 ng/sample) from FACS-purified normal and malignant T cells was assayed with the iScript One-step RT-PCR Kit with SYBR Green (Bio-Rad) using the aforementioned equipment. Reactions were run in triplicate. Results with *pol* and *env* were normalized to *elf2a*, assayed in parallel qRT-PCRs. Expression fold changes were calculated by the 2^−ΔΔCt^ method. Primers and reaction conditions were as follows:

Forward *pol* primer: CAG-CAC-AAA-CGA-AAA-TGG-TCT
Reverse *pol* primer: TGG-CTC-CTC-AGT-GTC-TCC-TT
Forward *env* primer: AGA-GGG-AAA-GGA-TGG-GAT-GT
Reverse *env* primer: TGT-TGG-ATG-TGG-TCT-GGT-CT
Forward *elf2a* primer: ATG-AGA-CAA-TGG-GGA-GAG-CA
Reverse *elf2a* primer: GGA-TGC-GGC-TGG-AGT-TTC
Denaturation: 95°C × 5 minutes
40 cycles:
  95°C × 10 seconds
  52°C × 10 seconds
  72°C × 30 seconds
Melt curve analysis—55°C–95°C.

### 2.7. Statistical Analyses

The student's *t*-test was used to compare differences in genomic relative copy number or fold changes in RNA expression. *P* values <0.05 were considered significant.

## 3. Results and Discussion

 We previously performed an ENU-mutagenesis phenotypic screen designed to identify abnormal T-cell phenotypes. Our screen resulted in the discovery of three *D. rerio* lines (*srk*, *hlk*, *otg*) prone to T-cell malignancies, specifically T-ALL and T-LBL [6]. To investigate non-germline acquired genetic changes occurring in these cancers, we used aCGH to compare DNA of neoplastic and normal tissues from individual fish of each of these lines. These experiments revealed several homologous genes that are commonly amplified or deleted in both zebrafish and human T-ALL [8].

**Figure 1:**
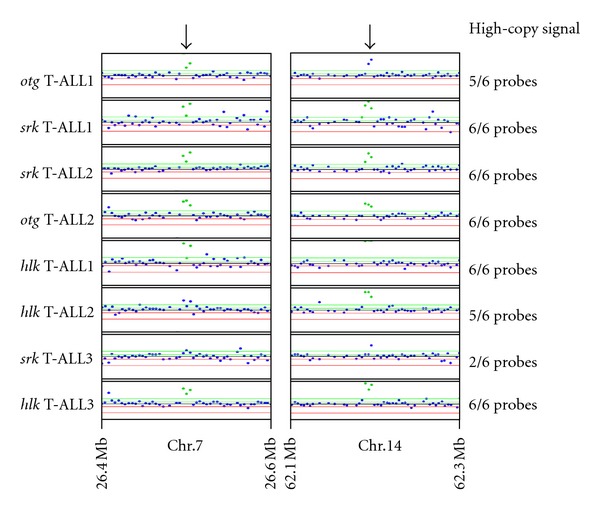
Recurrent amplifications of a small genomic locus in zebrafish T-ALL. Ten kb loci on chromosomes 7 and 14 (Zv6 genomic assembly) show high-copy gains with multiple probes in these regions (black arrows) of 8/8 *D. rerio* T-ALL samples tested. Signal intensities above a “high-copy gain threshold” (upper green line) indicate a greater than 2-fold increase in copy number. Individual probes and their intensities are depicted as blue dots; areas with 3 adjacent probes above the high-copy gain threshold use green dots to denote those probes. Seven cancers exhibited high-copy signals for ≥5/6 probes in this region, while *srk* T-ALL3 had high-copy signals for only 2/6 probes.

 In those studies, >98% of *D. rerio* genes with somatically acquired CNAs also had identifiable human counterparts. However, two non-homologous genomic regions unique to zebrafish were also particularly interesting. These loci showed copy number gains in 8/8 zebrafish T-cell cancer genomes relative to DNA of nonmalignant tissues from the same animals (Figure 1). Notably, T-ALLs from all three lines (3/3 *srk*, 3/3 *hlk*, 2/2 *otg*) exhibited copy number gains in both regions, establishing these acquired genomic amplifications as consistent features in T-cell cancers arising from different genetic backgrounds. Our aCGH experiments used a NimbleGen microarray platform constructed from the Zv6 genomic assembly. We subsequently discovered that the probes displaying amplified signals were mistakenly assigned to distinct regions on chromosomes 7 and 14 (hybridization data depicted in Figure 1). However, upon closer inspection these probes actually derive from a single, approximately 11 kb, locus. Intriguingly, this region corresponds to a genomically integrated retroviral element dubbed ZFERV by Shen and Steiner, named as such because it is the first and thus far only described zebrafish endogenous retrovirus [11].

**Figure 2:**
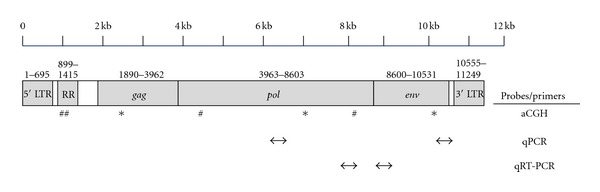
ZFERV Genomic Organization. The ZFERV retrovirus is comprised of two 695 bp long terminal repeats (LTRs), a 517 bp repeat region (RR) containing 9 direct repeats, and ORFs for 3 proteins: *gag*, *pol*, and *env* [11]. The *gag* and *pol* genes share the same reading frame and are predicted to be translated from one transcript by read-through of a stop codon; *env* uses a different reading frame and is probably a distinct transcript [11]. aCGH probe sites are shown beneath the ZFERV genome schematic: probes found on Zv6 chromosomes 7 (*) and 14 (^#^) are dispersed throughout ZFERV. One aCGH probe had sequences corresponding to the RR (^##^), and has multiple binding sites in this area. The 5 remaining aCGH probes map to ORFs. Amplicons from qPCR and qRT-PCR assays are also depicted (not shown to scale). The *pol* and *env* qPCR products are 168 and 169 bp, respectively; qRT-PCR products are 225 bp for *pol* and 234 bp for *env*.

 In scrutinizing the six aCGH probe sequences localized to these two chromosomes, we realized they were in fact distributed throughout the ZFERV genome (Figure 2). Collectively, our hybridization results with these 6 probes provide compelling evidence that the entire ZFERV locus is undergoing somatic amplifications in the genomes of zebrafish T-cell cancers. Because our aCGH data is internally normalized by comparing each cancer's DNA to paired non-malignant tailfin DNA from the same fish, our results are protected from possible ZFERV copy number variation (CNV) that might exist between different animals. However, due to ambiguity regarding initial (i.e., germline) ZFERV copy number in individual fish, it is impossible to deduce the absolute number of copies gained by each cancer. Instead, our findings are limited to the conclusion that ZFERV has been amplified, relative to the original number of ZFERV copies, in 8/8 T cell malignancies tested. Moreover, because “normal” ZFERV copy number and genomic locations may vary between fish or between strains, thus far, determining absolute ZFERV copy number prior to oncogenesis has been challenging.

 Reinforcing the complexity of this issue, previous *D. rerio* genome builds have displayed ZFERV in multiple locations on each assembly, and also on several different linkage groups (LG 1, 5, 7, 14, 15, 16, 17, and 22). It is inherently difficult to accurately map multicopy loci like ZFERV, and this is made even more taxing by its sequence composition. ZFERV harbors several redundant sequence tracks, including 5′ and 3′ long terminal repeats (LTRs) and a 517 bp repeat region (RR) containing 9 consecutive repeat elements (see Figure 2). When compounded with potential variability resulting from strain-specific ZFERV integrations, it is perhaps predictable that ZFERV has not received definitive chromosomal map position(s). Consequently, the current NCBI zebrafish genome actually suppresses ZFERV sequences and curates them so they do not appear on the Zv9 assembly at all.

 In the original report describing ZFERV, Shen and Steiner conducted studies to address some of these questions concerning copy number and genomic localization: to prove that ZFERV was integrated into the *D. rerio* germline, they tested sperm DNA from several Tübingen (Tü) fish and verified an integration site common to each of their genomes [11]. Additionally, using Southern blots of Tü genomic DNA, they detected 2–4 bands hybridizing to a ZFERV *env* probe, implying a maximum of four retroviral copies per haploid genome [11]. However, not all Tü fish showed identical hybridization patterns. This could be due to restriction site polymorphisms in the Tü strain but might also suggest that different fish, even from the same strain, can possess different ZFERV copy number and integration sites. Moreover, when Southern hybridizations with an LTR-based probe were performed, 8–10 bands were seen. Most—but not all—of these entities were shared by different Tü fish [11]. As with prior results, this finding might be attributable to variability in ZFERV copy number and genomic position between different fish. Another interpretation that must be considered is that homologous LTRs from other related retroviruses and/or incomplete ZFERV proviral genomes (having ≥1 LTR, but no *env*) would yield a similar experimental outcome.

In spite of these uncertainties, our aCGH data remain convincing as evidence of somatically acquired ZFERV amplifications in *D. rerio* T-cell cancers. None of our aCGH probes correspond to LTR sequences, and 5/6 derive from the retroviral *gag*, *pol*, or *env* genes (Figure 2). Furthermore, even if repeat elements had been used in hybridizations, our method of comparing neoplastic to non-malignant DNA from the same animal is designed to normalize for CNV discrepancies between different fish. Therefore, we conclude that zebrafish T-cell malignancies acquire non-germline ZFERV copies at some point after fertilization, but whether amplifications precede and contribute to oncogenesis is unclear.

Because ZFERV transcription occurs in normal zebrafish T cells [11], we were curious whether normal *D. rerio* T lymphocytes might also have ZFERV copy gains. To determine if retroviral amplifications also occur in nonleukemic T cells, we investigated ZFERV in normal zebrafish T lymphocytes. To emulate our aCGH comparisons, we developed quantitative PCR (qPCR) assays for two ZFERV genomic regions. Using DNA from cancers with gains identified by aCGH, we verified these assays' ability to detect ZFERV copy number gains (data not shown). Next, we employed these qPCRs of amplicons from the *pol* and *env* regions (locations shown in Figure 2) to test genomic DNA from tailfin tissue and FACS-purified T cells of wild-type (WT) adult zebrafish. Thymocytes were obtained from WT WIK *D. rerio* carrying an *lck*::*EGFP* transgene [12]. Since the zebrafish *lck* promoter is T cell specific, T lymphocytes from this line are GFP^+^. However, unlike fish with T-ALL or T-LBL, adult (>6 months of age) WT fish have significantly fewer T cells (approximately 5 × 10^4^ GFP^+^ thymocytes/fish; our unpublished observations). Consequently, we pooled thymic tissue from several WT fish for FACS purifications. We then analyzed amplicons from both ZFERV regions to independently assay copy number differences.

**Figure 3:**
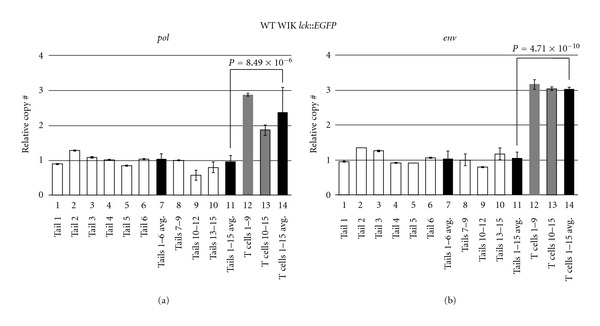
ZFERV amplifications in WT WIK *D. rerio* T cells. Genomic DNA from 15 WIK *lck::EGFP* fish was analyzed by qPCR of the ZFERV *pol* (a) and *env* (b) genes. White bars depict results from tail DNA of individual fish (lanes 1–6) or groups of 3 fish (lanes 8–10). Black bars show calculated means of 6 singly tested tails (lane 7) or all 15 tails (lane 11). “Tails 7–9” sample (lane 8) was arbitrarily assigned copy number equal to 1, and this DNA was used as the reference standard for all subsequent qPCRs. T cells pooled from 9 or 6 WT fish (gray bars) had 2- to 3-fold gains in ZFERV. Mean copy number was higher in T cells than tailfin DNA for the 15 fish cohort (lane 11 versus 14). Zebrafish *elf2a* (1 copy/haploid genome) qPCR was used to normalize *pol* and *env* results (not shown). Water-only template controls lacked detectable product (not shown). Reactions were performed in triplicate, and error bars show standard deviations (*env* qPCR of tails 2 and 5 had standard deviations too small to be seen).

Tailfin DNAs were tested individually or in small groups to ascertain whether there were appreciable germline CNV differences in WIK strain fish (Figure 3, lanes 1–6 and 8–10). As seen in these data, qPCR of *pol* (Figure 3(a)) and *env* (Figure 3(b)) show little deviation between fin DNA from different WIK fish, implying that CNV was minimal in these strain-related animals (lanes 7 and 11). Because copy number was so uniform, this further suggests that ZFERV amplification does not occur in fin tissue. Thus, we conclude that ZFERV status in fin tissue likely represents true germline copy number, and that this level is relatively stable between individual fish.

In contrast, normal T cells pooled from these same WT fish showed significant ZFERV gains relative to tailfin DNA (Figure 3, lanes 12, 13). On average, WIK T cells had 2- to 3-fold as many ZFERV copies as matched tail DNA (compare lane 11 to 14). Since germline copy number is unknown, we cannot deduce the real number of ZFERV copies in these T cells. Nonetheless, if prior data suggesting 2–4 copies/haploid genome are accurate [11], these results indicate normal T cells may average up to 12 copies per haploid genome, or 24 copies/diploid T cell. If correct, this would compute to 16 new ZFERV integrations, on average, in each T cell.

Because we used T lymphocytes pooled from several WIK fish in these studies, we cannot definitively conclude whether all animals' T cells bore evidence of ZFERV amplification. It is possible that only one or a few fish have ZFERV gains, with DNA from those fish skewing the average upward. However, even in one fish, T lymphocytes constitute a nonclonal population. It is possible—perhaps even likely—that ZFERV copy number varies on a cell-to-cell basis. ZFERV amplifications may occur in T cells themselves; alternatively, they might take place earlier along the hematopoietic stem cell/T cell progenitor differentiation spectrum. We have not tested precursor populations, as these are impossible to obtain in *D. rerio* owing to the dearth of antibodies to cell surface receptors. Irrespective of its precise timing, we conclude that thymocytes acquire additional genomic ZFERV copies at some point after fertilization, exactly like those detected in our aCGH analyses of zebrafish T-cell cancers.

Notably, there is precedent proving that ZFERV is active in zebrafish T cells. This retrovirus was originally discovered from a thymic cDNA library, after adult *D. rerio* thymus had been subtracted against 2-day postfertilization (dpf) larval fish, which have not yet developed T lymphocytes [11]. This study identified 43 clones hybridizing to only adult thymic cDNA. Of these, 21 clones also showed thymus-specific staining in 7-dpf *in situ* hybridizations (ISH). After sequencing, Shen and Steiner recognized that all 21 clones derived from various segments of the ZFERV genome [11]. So, not only was ZFERV transcribed by both 7-dpf and adult thymocytes, its expression in these cells was significantly higher than in other tissues by these two methodologies. Subsequent ISH experiments in 4-dpf, 5-dpf, and 3-month-old juvenile fish, as well as Northern blotting of RNA from adult fish thymocytes, confirmed these findings [11]. Together, these prior studies and our own new findings demonstrate that ZFERV is highly transcribed by larval and adult *D. rerio* thymocytes and that ZFERV amplifications occur in the genomes of normal and malignant zebrafish T cells.

To further expand our understanding of these phenomena, we next compared ZFERV amplifications in cancer-prone thymocytes and neoplastic T cells to WT T cells. For these experiments, we used our qPCR assays to compare ZFERV copy number in two other T-cell malignancy predisposed lines, *hlk* and *MYC-ER*. Both of these lines are prone to T-LBL and T-ALL, allowing ZFERV quantification of their germlines, their “premalignant” T lymphocytes, and their malignant T cells. All *MYC* transgenic fish have hypertrophic thymi, likely reflecting abnormal T-cell proliferation and physiology. In contrast, *hlk* fish carry an unidentified mutation, display normal-appearing thymi, and the molecular basis for their cancer predisposition is unknown. T-ALL or T-LBL afflicts roughly 35% of *hlk* homozygotes by one year [6], reflecting a requirement for additional mutations to promote malignant transformation [8]. By comparison, WT *lck*::*EGFP* fish rarely develop T-cell cancers (<0.1%, our unpublished observations) and have normal T-cell development and physiology [12]. Thus, using these samples we could investigate whether normal, abnormal, and neoplastic T cells all exhibited similar degrees of ZFERV amplification.

**Figure 4:**
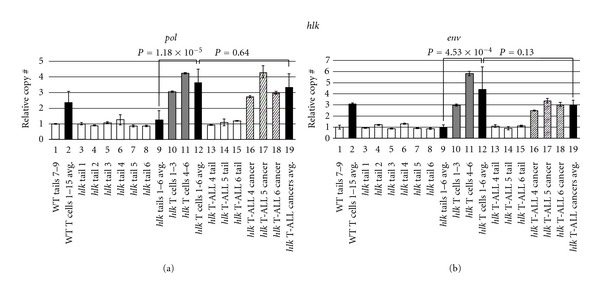
ZFERV amplifications in *hlk* zebrafish. DNA from 6 *hlk* fish with normal phenotype and 3 with GFP^+^ cancers was tested by qPCR of *pol* (a) and *env* (b). White bars show tail DNA of individual normal (lanes 3–8) or T-ALL^+^ (lanes 13–15) fish. All were statistically similar to each other and to WT Tails 7–9 (lane 1). Pooled T cells from *hlk* fish without T-ALL (gray bars) had ZFERV gains comparable to normal WIK T cells (lane 2). Mean copy number was higher in *hlk *T cells than tails (lane 9 versus 12) in the same animals. Diagonally striped bars show amplifications in neoplastic T cells of 3 *hlk* fish (lanes 16–18). Mean copy gains were similar in non-malignant and malignant *hlk* T cells (lane 12 versus 19). Other details are as described in the legend to Figure 3.

**Figure 5:**
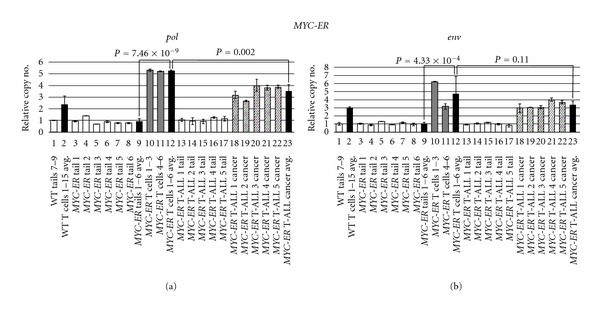
ZFERV amplifications in *MYC-ER* zebrafish. Phenotypically normal (*n* = 6) or T-ALL^+^ (*n* = 5) *MYC-ER* fish were tested by qPCR of *pol* (a) and *env* (b). White bars display tail DNA from single normal (lanes 3–8) or diseased (lanes 13–17) fish. *MYC-ER* tails had similar copy number to each other (compare lanes 3–8 and 13–17) and to WT Tails 7–9 (lane 1). T cells pooled from groups of 3 normal *MYC-ER* fish (gray bars) showed ZFERV amplification; higher gains were seen in *MYC-ER* than WT T cells (lane 2 versus 12; *P* values 5.86 × 10^−4^ for *pol*, 0.15 for *env*). T cells showed 4- to 5-fold higher ZFERV copy than matched tails (lane 9 versus 12). Diagonally hatched bars depict amplifications in T-ALL cells from 5 *MYC-ER* fish (lanes 18–22). Cancer ZFERV levels were well above paired tails (compare lanes 13–17 to 18–22). Slightly lower gains were seen in cancerous than non-malignant *MYC-ER* T cells (lane 12 versus 23); this reached statistical confidence for *pol*, but not *env*. Other details are as listed in Figure 3's legend.

As in earlier experiments, we examined tailfins from individual fish to ascertain ZFERV germline variability. Tails from single *hlk* and *MYC-ER* fish (Figures 4 and 5, lanes 3–8) demonstrated consistent copy number between animals. Moreover, both *hlk* and *MYC-ER* tails had ZFERV CNV similar to the WT WIK line (compare lane 1 to other white bars in Figures 4 and 5). Based on these results, identical for both the *pol* and *env* regions, we conclude that all 3 lines have approximately equivalent germline copies of ZFERV. In pooled premalignant T cells (i.e., thymocytes from *hlk* and *MYC-ER* fish lacking tumors or other non-thymic GFP), genomic ZFERV was again elevated relative to tailfin DNA from the same fish (Figures 4 and 5, compare gray bars in lanes 10-11 to white bars in lanes 3–8). Overall, mean T-cell ZFERV copy number was roughly 3-fold above germline in WT, 4-fold higher in *hlk*, and 5-fold increased in *MYC-ER* (compare lane 9 to 12 in both figures). Since WT, *hlk*, and *MYC-ER* thymocytes all showed approximately equivalent gains, we infer that genomic integration is not appreciably enhanced in T lymphocytes of either cancer-prone genotype. Thus, while retroviral amplification is clearly a common feature of all *D. rerio* T cells, cancer predisposition probably does not directly originate from increased susceptibility to ZFERV integration, as these events evidently transpire in normal T cells regularly. However, it is plausible that cancer predisposing mutations and ZFERV copy number gains may cooperate to promote malignant transformation of T cells, as amplifications were uniformly present in every T-ALL sample examined by aCGH.

To investigate how WT and cancer-prone T cells compare to actual neoplasias in the *hlk* and *MYC-ER* lines, we also analyzed malignancies from these same genetic backgrounds. We performed *pol* and *env* qPCRs on 3 *hlk* and 5 *MYC-ER* fish, each of which had large thymic tumors and/or extensive GFP^+^ disease in extra-thymic areas (Figures 4 and 5). Tail DNA from these 8 fish all had similar germline ZFERV content to previously tested tailfin samples (compare lanes 13–15 in Figure 4 and lanes 13–17 in Figure 5 to other white bars in both figures). Like *hlk* T lymphocytes, *hlk* cancers had ZFERV amplification (Figure 4, lanes 16–18). However, gains were similar in magnitude to those seen in *hlk* premalignant T cells (compare lane 12 versus 19). In *MYC-ER* cancers, ZFERV gains were also detected (Figure 5, lanes 18–22). As in *hlk*, benign and malignant *MYC-ER* T cells did not show appreciable copy number differences (compare lane 12 to 23).

**Figure 6:**
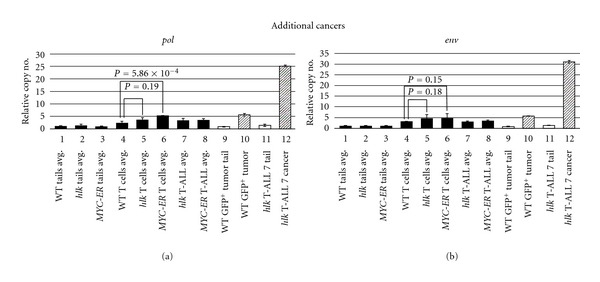
ZFERV amplifications in two other *D. rerio* T-cell cancers. One WT WIK fish spontaneously developed a GFP^+^ thymic tumor and was tested by qPCR of *pol* (a) and *env* (b). Average copy numbers of other samples tested previously are shown as black bars. Germline ZFERV copy number in this fish (lane 9) was similar to the 15 WIK fish already examined (lane 1). This tumor showed 5.5-fold amplification (lane 10, diagonal bar), similar to non-malignant T cells and cancers from WT, *hlk*, and *MYC-ER* fish (lanes 4–8). One other *hlk* T-ALL exhibited high-level, 25- to 30-fold gains (lane 12), although its germline copy number (lane 11) was comparable to other fish (lanes 1–3).

We also tested 2 other malignancies by qPCR. In one WT *lck*::*EGFP* fish, we noticed a large GFP^+^ thymic tumor. In our experience, the spontaneous occurrence of T-cell cancer in WT fish is exceedingly rare, so we used this opportunity to investigate whether ZFERV amplification accompanied this event. Tail DNA indicated this animal had normal ZFERV germline content (Figure 6, compare lane 1 versus 9), and cancerous T cells from this fish showed approximately 5.5-fold higher copy number (lane 10). This degree of amplification is roughly twice that seen in normal WIK T cells and more closely resembled typical copies in *MYC-ER* T cells and cancers (compare lane 10 to lanes 4, 6, and 8). However, since this result reflects only one tumor, no general conclusions can be drawn about retroviral amplification in the rare cancers of WT fish. Lastly, in one additional *hlk* cancer, we found remarkably high ZFERV levels, showing 25- to 30-fold amplification above germline (lanes 11 and 12). This degree of copy number gain is nearly ten times higher than the other 9 T-ALLs we examined by qPCR, or the 8 tested previously by aCGH. Nonetheless, this infrequent scenario clearly demonstrates that ZFERV can parasitize the zebrafish genome in striking fashion, as this cancer likely harbors as many as 50–100 newly acquired retroviral copies.

Taken together, we conclude that virtually all MYC-driven, *hlk*-, *srk*-, and *otg*-induced, or even spontaneous zebrafish T-cell cancers have ZFERV amplifications. However, since nearly all benign, cancer-prone, and malignant T cells show similar genomic levels, the absolute amount of ZFERV amplification does not appear to be an important oncogenic determinant. This is not surprising, as it is likely that the site rather than the number of integrations is the crucial factor. To pursue this premise, one could identify new loci where ZFERV has integrated into cancer genomes, with the hypothesis that these might lie near or within proto-oncogenes or tumor suppressors. We have initiated such studies, and they are currently in progress. As an adjunct, we chose to investigate transcription of ZFERV-derived RNAs. We reasoned that integrations into transcriptionally permissive genomic sites might be accompanied by increased ZFERV RNA expression, perhaps signifying “active” proviral copies. While these insertions might not denote sites where oncogenes or tumor suppressor reside, it could serve as a proxy for ZFERV promoter potency in the genome overall. If so, this predicts that cancers would have higher ZFERV transcription than normal T cells and perhaps premalignant T lymphocytes as well.

**Figure 7:**
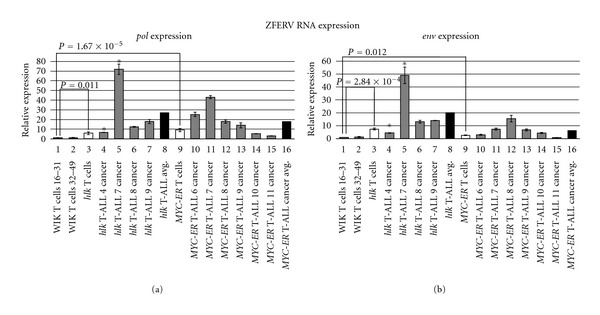
ZFERV gene expression by normal and abnormal *D. rerio* T cells. Total RNA was tested by qRT-PCR of the ZFERV *pol* (a) and *env* (b) genes. Normal T cell RNA pooled from WIK fish (*n* = 16 and 18; lanes 1, 2) were used as control, and the “WIK T cells 16–31” sample was arbitrarily set to an expression value = 1. Premalignant T lymphocytes from *hlk* (*n* = 6) and *MYC-ER* (*n* = 3) fish had higher expression than WT fish (white bars; lanes 3, 9), and this higher transcription reached statistical significance. Individual cancers from *hlk* (*n* = 4; lanes 4–7) and *MYC-ER* (*n* = 6; lanes 10–15) fish are depicted with gray bars. Cancer cells from these fish invariably showed higher *pol* and *env* transcripts than T cells from WT fish, and nearly always had elevated RNA expression relative to normal T cells from these same two lines. Mean expression of *pol* and *env* in malignant T cells (black bars; lanes 8, 16) exceeded both WT and premalignant T cell transcript levels. Two cancers highlighted by asterisks (*hlk* T-ALL 4, *hlk* T-ALL 7; lanes 4, 5) were also tested for genomic copy number by qPCR. The *hlk* T-ALL 4 cancer had ZFERV copy number similar to *hlk* premalignant T cells (see Figure 4), and its *pol* and *env* expression also resembled *hlk* T cells. Cells from the *hlk* T-ALL 7 sample had high-level genomic ZFERV gains (see Figure 6), and likewise demonstrated dramatically increased ZFERV transcription.

To conduct these studies, we developed quantitative Reverse Transcription-Polymerase Chain Reactions (qRT-PCR) of the ZFERV *pol* and *env* genes (amplicon locations shown in Figure 2). As for qPCR, we used pooled normal T cells from WT WIK fish as our reference. Recall that even normal T lymphocytes highly express ZFERV transcripts [11], so these RNAs are already plentiful in the cells used as our standard. Results for *pol* and *env* were highly reproducible between two pooled T-cell samples from different groups of WT fish, and this value was arbitrarily assigned an expression level of 1 (Figure 7, lanes 1 and 2). By comparison, pooled pre-cancerous T cells from *hlk* fish exhibited approximately 6-fold and 7-fold enhanced *pol* and *env* transcription, respectively (lane 3, white bar). Likewise, pooled premalignant T cells from *MYC-ER* fish also had higher ZFERV transcripts (lane 9; *pol*: 9-fold increase, *env*: 2.5-fold increase). So, while ZFERV genomic amplification did not differ impressively between WT and cancer-prone T cells (3- to 5-fold; Figure 6), expression of retroviral transcripts was more pronounced in T cells from both cancer-prone genotypes.

We also examined T-cell cancers from both lines (*n* = 10; 4 *hlk*, 6 *MYC-ER*). In *hlk* malignancies, all 4 cancers (lanes 4–7, gray bars) showed increased *pol* and *env* compared to normal T cells. One cancer (*hlk* T-ALL 4; lane 4) resembled premalignant *hlk* T cells in its transcriptional profile. This same tumor had also been tested by qPCR and showed comparable ZFERV amplification to non-malignant *hlk* T cells (see Figure 4, lanes 12 and 16). So, in this instance, copy number mirrored ZFERV expression. Three other *hlk* cancers (Figure 7, lanes 5–7) had greater *pol* and *env* transcription than *hlk* premalignant T cells, with at least 2-fold increases in both transcripts. One of these (*hlk* T-ALL 7; lane 5) had markedly higher levels, with 13-fold *pol* upregulation and 7-fold higher *env* than non-malignant *hlk* T cells (lane 3). The *hlk* T-ALL 7 sample was also analyzed by qPCR and exhibited high copy gains (Figure 6, lane 12), providing a second example that correlated genomic copy number to ZFERV transcriptional activity. Overall, mean transcription was 5-fold greater for *pol* and 3-fold higher for *env* in *hlk* cancers than their pre-cancerous T lymphocytes (compare lane 3 versus 8), although the *hlk* T-ALL 7 cancer skews this result somewhat. That notwithstanding, every *hlk* cancer showed ≥4-fold upregulation of both transcripts relative to WT thymocytes, proving that higher ZFERV expression does coincide with malignancy.

Similar findings were also obtained in 6 *MYC-ER* cancers (lanes 10–15, gray bars). Although transcript levels varied in individual cancers, mean *pol* expression was 2-fold increased and *env* was 3-fold higher in all six malignancies compared to *MYC-ER* premalignant T cells (compare lane 9 versus 16). Expression of *pol* and *env* by the same tumor usually followed the same trend. However, some cancers did have discordant transcription of these two genes. Despite these disparities, *MYC-ER* cancers averaged 18- and 7-fold higher *pol* and *env*, respectively, than normal T cells from WT fish, further implicating ZFERV in zebrafish T-cell oncogenesis.

Though ZFERV copy number and transcriptional activity correlated in the two cancers where we evaluated both genomic and expression data, variation between *pol* and *env* in the same tumor requires another explanation. Unlike ZFERV *gag*-*pol*, which is thought to be transcribed as a single RNA, *pol* and *env* come from distinct transcripts [11]. Thus, these genes could be differentially regulated. In addition, other factors may impact overall ZFERV transcription. As noted previously, certain integration sites might foster retroviral activity. In addition, cancers with very high copy number might be expected to have commensurate RNA levels, and our limited data support this. Another potential factor regulating transcription pertains to normal patterns of ZFERV expression in T lymphocytes. While it is known that *D. rerio* T cells normally make ZFERV RNA ([11] and this work), it is not known if all T-lineage cells do, or rather if only some T lymphocyte developmental stages have active ZFERV. Since T-ALL can exhibit differentiation arrests at multiple maturational stages [13, 14], it is possible that individual cancers with differing arrest points might also demonstrate different ZFERV transcription patterns. Unfortunately, the lack of antibody reagents able to recognize zebrafish T cell surface markers currently limit testing of this latter hypothesis.

Despite these limitations, our findings indicate that both genomic amplification and transcription of ZFERV may impact normal *D. rerio* T-cell biology and oncogenesis. In particular, our results bolster the notion that new retroviral integrations could be pathologic on the molecular level. Stably integrated retroviral elements are common in vertebrate genomes, with nearly 10% of the human genome comprised of ERVs or their derivatives [15]. However, most ERVs are inert due to their accrual of point mutations and partial deletions. ZFERV is atypical in that its genes apparently retain unmutated ORFs. Moreover, these genes are robustly transcribed by *D. rerio* T cells as verified by ISH, Northern blotting, and qRT-PCR ([11] and this paper). The abundance of ZFERV RNA in T lymphocytes is perhaps not surprising, as ZFERV's LTR was the most potent promoter among several transcriptional regulatory sequences assayed in a carp (*Cyprinus carpio*) epithelial cell line, including the oft-used CMV promoter [16].

Rather, ZFERV's apparent T-cell specificity may be the more intriguing finding. Shen and Steiner identified putative binding sites for the lymphoid transcription factors Ikaros and Tcf3 (E47) in the ZFERV LTR, but also for other factors (FOS/JUN, C/EBP, STAT, NF-*κ*B, and others) that are more general activators of transcription [11]. Indeed, the sequencing of ZFERV-derived transcripts by EST projects from several other tissue types suggests that non-T-cells may transcribe ZFERV also [11]. Whether this finding reflects low-level ZFERV transcription by other cell types, or low-level T-cell contamination in these tissues, is not clear. In either case, the atypical persistence of intact ZFERV ORFs, and their transcriptional activity in zebrafish T cells, raises the question of whether ZFERV proteins might serve a functional purpose. Selective pressure would normally favor mutations disabling a potentially genotoxic retrovirus. Instead, we hypothesize that ZFERV may in fact serve some important biologic role, accounting for its paradoxical maintenance as an active retrovirus in the zebrafish genome.

ZFERV's apparent absence in the genomes of other *Danio* genera [11] implies that its entry into zebrafish is fairly recent in evolutionary terms, but ZFERV sequences have been identified from several different strains, suggesting that its integration is pervasive in the species. It is not known whether ZFERV is present in all *D. rerio*, and to our knowledge, this question has not been investigated. To date, the closest relative to ZFERV is an exogenous piscine retrovirus, SSSV. Curiously, this virus is linked to swim bladder leiomyosarcomas in Atlantic salmon, and like our results with ZFERV, these tumors show high copy number proviral SSSV integration [17].

Besides its close relation to SSSV, ZFERV also shares sequence conservation and similar genomic structure with gammaretroviridae of the murine leukemia virus (MLV) class [11, 17]. MLV-related retroviruses are known to be oncogenic by insertional mutagenesis [18], and the determinants governing their preferred integration sites have been the subject of intense scientific scrutiny [19–21]. Although an obvious ZFERV homologue has not been identified in humans, other MLV-related sequences have been detected in human cell lines. However, it appears that these retroviral sequences may have been acquired by human cells during xenografting into murine recipients or result from reagent contamination by murine DNA [22, 23]. In addition, a long ORF on human chromosome 14 bears high homology to ZFERV's *env*, and upstream sequences contain a short *gag*-*pol* element [24]. So, ZFERV-related retroviruses are evidently integrated in the human genome as well. Incorporating all these circumstantial data, it becomes plausible that ZFERV integrations—like SSSV and MLV—may not only be oncogenic in zebrafish, but might also have relevance for human biology in general.

## 4. Conclusions

 Nearly a decade ago, Shen and Steiner discovered a zebrafish endogenous retrovirus, ZFERV, based on its high transcriptional activity in larval and adult *D. rerio* thymocytes [11]. Their work suggested that multiple copies of ZFERV existed in the zebrafish genome, and since that time, the loci where ZFERV resides still have not been definitively assigned. These difficulties are probably attributable to the fact that this multicopy locus may vary in copy number and genomic positioning in different fish. Amidst this backdrop, we have found that ZFERV copy number is increased still further in every *D. rerio* T-cell malignancy we examined from 4 different genetic lines.

Somewhat surprisingly, our results demonstrate that ZFERV amplification is not unique to cancerous T cells. Rather, copy number gains also occur in T lymphocytes of WT *D. rerio*, the same cells where ZFERV transcription was first identified. Moreover, ZFERV copy number appears to be fairly consistent amongst normal, premalignant, and malignant T cells (Figure 6, lanes 4–8), although individual cancers can occasionally show even higher levels of ZFERV in their genomes. It is possible that individual normal T cells have similar variability in ZFERV copy number, but this has not been experimentally addressed.

As seen with genomic amplifications, ZFERV transcription occurs within normal, pre-cancerous, and neoplastic T cells. Our results suggest that expression of ZFERV RNAs is higher in cancer samples, but we do not recognize a consistent trend from one cancer to the next. Nonetheless, these commonalties between normal and malignant T lymphocytes imply that ZFERV activation and amplification may be a normal feature of zebrafish T cell biology, with no pathologic consequence. Still, ZFERV's abundant transcription, apparently functional ORFs, and ability to undergo genomic amplification all allude to its oncogenic potential. Compounded with mutations like *hlk*, *srk*, and *otg* that confer malignancy predisposition, ZFERV may help promote T-cell transformation. Given that the closest phylogenetic relatives of ZFERV are an exogenous piscine retrovirus linked to sarcomagenesis and MLV-class retroviruses that are leukemogenic via genomic integration, it is tempting to speculate that ZFERV may contribute to cellular immortalization by similar mechanisms.

Certainly, ZFERV integrations in crucial genomic sites could have transformative properties. For example, integration within a tumor suppressor gene might render it unable to generate its normal protein product, thereby ablating function. Conversely, integrations into the promoter or enhancer regions of proto-oncogenes might augment their transcription. Since ZFERV appears to be specifically and highly expressed by thymocytes, this scenario could be analogous to the translocation of proto-oncogenes into the T-cell receptor loci, which are well described in T-ALL [25, 26]. However, proof of this hypothesis will require identification of somatically acquired ZFERV integrations at these genomic sites. At this point, the possibility that ZFERV amplifications may contribute to T-cell oncogenesis remains an open question that will require further investigation to resolve decisively.
